# RETRACTION: Design and development of thyroxine/heparin releasing affordable cotton dressings to treat chronic wounds

**DOI:** 10.1155/term/9795348

**Published:** 2025-08-06

**Authors:** Journal of Tissue Engineering and Regenerative Medicine

RETRACTION: T. S. Waris, S. T. Abbas Shah, A. Mehmood, et al., “Design and development of thyroxine/heparin releasing affordable cotton dressings to treat chronic wounds,” *Journal of Tissue Engineering and Regenerative Medicine* 2022 (2022): term.3295, https://doi.org/10.1002/term.3295.

The above article, published online on 4 March 2022 in Wiley Online Library (https://wileyonlinelibrary.com), has been retracted by agreement between the journal's Chief Editor Catherine K. Kuo and John Wiley & Sons Ltd.

The retraction has been agreed following an investigation of the concerns raised by *Mycosphaerella arachidis* on PubPeer [1], which identified unexpected similarities between multiple panels of Figure 6(a).

More specifically, the images depicting the wounds of animals in the Hep-W and Control-W groups after 20 days appear identical to the image of the T4-W group after 15 days. Further concerns have also been identified related to the similarity of two panels to images shown in Figure 7 from another publication by the author group, where they are attributed to a different experimental condition [2].

As a result of the investigation, the data and conclusions of this article are considered unreliable.

The authors disagree with this retraction.
